# Modeling and Implementation of Multi-Position Non-Continuous Rotation Gyroscope North Finder

**DOI:** 10.3390/s16091513

**Published:** 2016-09-20

**Authors:** Jun Luo, Zhiqian Wang, Chengwu Shen, Arjan Kuijper, Zhuoman Wen, Shaojin Liu

**Affiliations:** 1 Changchun Institute of Optics, Fine Mechanics and Physics, Chinese Academy of Sciences, Changchun 130033, China; luojun604@sina.com (J.L.); shenchengwu@ciomp.ac.com (C.S.); wenzhuoman@gmail.com (Z.W.); evsv@sohu.com (S.L.); 2 University of the Chinese Academy of Sciences, Beijing 10049, China; 3 Fraunhofer Institute for Computer Graphics Research, Darmstadt 64283, Germany; arjan.kuijper@igd.fraunhofer.de; 4 Mathematical and Applied Visual Computing, Technische Universität Darmstadt, Darmstadt 64283, Germany

**Keywords:** orientation, north finding, azimuth, gyro misalignment, shaft misalignment, uncertainty analysis

## Abstract

Even when the Global Positioning System (GPS) signal is blocked, a rate gyroscope (gyro) north finder is capable of providing the required azimuth reference information to a certain extent. In order to measure the azimuth between the observer and the north direction very accurately, we propose a multi-position non-continuous rotation gyro north finding scheme. Our new generalized mathematical model analyzes the elements that affect the azimuth measurement precision and can thus provide high precision azimuth reference information. Based on the gyro’s principle of detecting a projection of the earth rotation rate on its sensitive axis and the proposed north finding scheme, we are able to deduct an accurate mathematical model of the gyro outputs against azimuth with the gyro and shaft misalignments. Combining the gyro outputs model and the theory of propagation of uncertainty, some approaches to optimize north finding are provided, including reducing the gyro bias error, constraining the gyro random error, increasing the number of rotation points, improving rotation angle measurement precision, decreasing the gyro and the shaft misalignment angles. According them, a north finder setup is built and the azimuth uncertainty of 18” is obtained. This paper provides systematic theory for analyzing the details of the gyro north finder scheme from simulation to implementation. The proposed theory can guide both applied researchers in academia and advanced practitioners in industry for designing high precision robust north finder based on different types of rate gyroscopes.

## 1. Introduction

The rate gyroscope (gyro) north finder is an orientation instrument that finds the azimuth by measuring a projection of the earth rotation rate onto the gyro’s sensitive axis. It has been widely used in e.g., aiming techniques, surveying techniques, and navigation of autonomous ground vehicles [1,2,3]. It is also capable of providing the required azimuth reference information when the GPS signal is blocked, for instance in underground environments. Usually, north finding with an uncertainty of 206”(206” = 1 milliradian) is required in these applications [4]. However, more accurate north finding systems are always popular in practical application, e.g., if the aiming distance is 1 km, the aiming error is about 1 m when the azimuth uncertainty is 206”, and the aiming error is about 0.17 m when the azimuth uncertainty is 36” . Thus improving the north finding system precision is valuable to both applied researchers in academia and advanced practitioners in industry. In current gyro north finder systems, different types of gyros are used to measure the angular rate with respect to an inertial frame of reference, including Micro-Electro-Mechanical System (MEMS) gyros, ring laser gyros, fiber optic gyros, resonant optical gyros and dynamically tuned gyros [5]. According to their general usage categories, gyros can be sorted as consumer, tactical, navigational and strategic type. Recently, the structures of different gyros have been comprehensively analyzed to constrain the gyros’ bias error, scale-factor error and random error, e.g., MEMS gyros [6,7,8,9], ring laser gyros [10,11], fiber optic gyros [10,12], resonant optical gyros [13,14,15,16] and dynamically tuned gyros [17,18,19]. Other very active areas include constraining the gyro bias error and modeling of temperature drift through different kinds of filter methods [20,21,22,23,24,25,26]. However, only few works present the details of building a complete gyro north finder system that provides high precision azimuth reference information.

It is a challenge to design a high precision robust north finder system and to provide the corresponding azimuth measuring model. Many factors should be taken into consideration. Firstly, the model should take the gyro’s characteristics into account, including the gyro bias, the gyro random error and the scale-factor error. Secondly, the relationship of the gyro and shaft misalignment angles against the azimuth model needs to be clarified. The gyro misalignment angle is the mechanical install error between the shaft (rotation axis) and the gyro axis; the shaft misalignment angle is the tilt between the practical shaft and the vertical one. Finally, the azimuth measurement precision is affected by several elements such as the measurement points and encoder precision; thus these elements should be considered by the system model.

Currently, two of the most commonly used approaches to attain high precision robust azimuth results are two-point gyro north finding [27] and multi-point continuous rotation gyro north finding [4,28,29,30]. In the former case, the gyro is rotated by $180^{\circ }$. By subtracting the azimuth measurement results at the two positions, the additive bias error and scale-factor error is mitigated. However, it is not robust to the gyro random error. For achieving high azimuth measurement precision, the latter approach is more often used, because it is not only robust to the gyro bias error and the scale-factor error, but also to the random error. In particular, Prikhodko provides a comprehensive analysis about how the gyro misalignment, the gyro bias error, and the number of measurement points affect the north finding precision, but does not provide the azimuth measuring model [4]. Recently, a north finding system consisting of an uniaxial MEMS accelerometer and a fiber optic gyro is proposed, and a translocation method is adopted to eliminate sensor constant drift and scale factor error [31]. Bojja improves this system by replacing the uniaxial MEMS accelerometer with a two-axis MEMS accelerometer, and an indexing method is used to remove the sensor bias [32]. However, these methods still possess three shortcomings. The first one is that the gyro detects not only the earth rotation rate but also the turntable rotation rate due to the gyro misalignment and the shaft misalignment. The second one is that the vibration produced by the continuous rotation corrupts the gyro outputs. Finally, there exists no generalized mathematical model which analyzes the elements that affect the azimuth measurement precision. Hence, proposing an appropriate azimuth measuring scheme and providing the corresponding azimuth measuring mathematical model for designing and evaluating the north finder are urgently needed.

In this paper we propose a multi-position non-continuous rotation gyro north finder scheme to measure the azimuth between the observer and the north direction. We are the first to deduce the mathematical model of the gyro outputs against the azimuth at each rotation position explicitly. Our approach demonstrates the effects of the gyro and the shaft misalignments on the gyro outputs. Combining the gyro outputs model and the theory of propagation of uncertainty, we thus obtain an optimized azimuth measurement scheme.

The rest of the paper is organized as follows. Section 2 presents the gyro north finder structure and the multi-position non-continuous azimuth measuring scheme. Section 3 gives the gyro outputs model. The optimized north finding scheme is described in Section 4. Experimental results are discussed in Section 5. Finally, conclusions are drawn in Section 6.

## 2. The Multi-Position Non-Continuous Rotation Gyro North Finder Scheme

**Figure 1:**
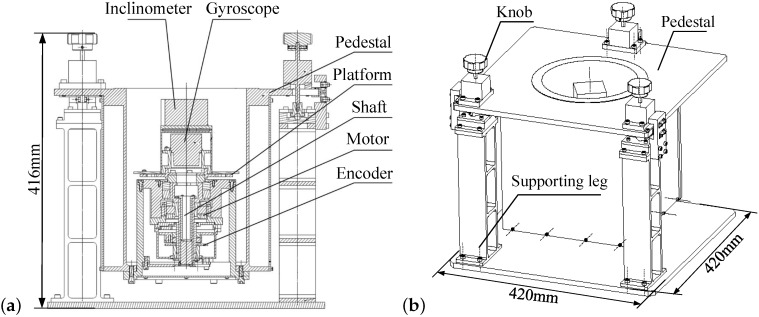
Layout of the north finder. (**a**) Inside part. (**b**) Outside part.

Figure 1 demonstrates the layout of the north finder whose length, width and height are 420 mm, 420 mm and 416 mm respectively. Inside a pedestal, an inclinometer, a gyro, a platform, a shaft, a motor, and an encoder (see Figure 1a) are placed. The inclinometer and the gyro are mounted on the platform that is fixed to the shaft. The pedestal is shored up by three supporting legs whose heights are adjusted by the three knobs (see Figure 1b).

These devices are divided into two categories according to their functions. The first category measures the shaft misalignment angle and aligns the shaft. It includes the inclinometer, the three knobs and the three support legs. The inclinometer is used to measure the angle of the platform with respect to the horizontal plane, and the shaft tilt is adjusted by the three knobs using the inclinometer outputs. Secondly, the gyroscope, the platform, the shaft, the motor, and the encoder are used to measure the earth rotation rate onto the gyro sensitive axis at each rotation position. The gyro detects the projection of earth rotation rate onto its sensitive axis. The motor controls the gyro (the platform and the shaft) to move non-continuously from position 1 to position *n*. The encoder records the rotation angle of the gyro.

**Figure 2:**
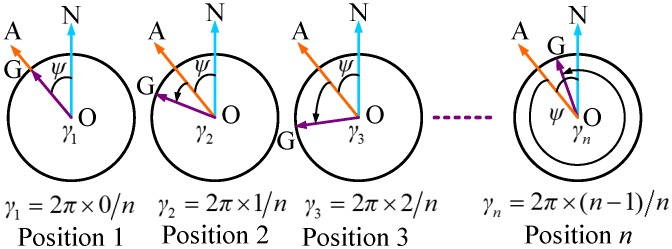
The gyro north finder multi-position non-continuous rotation scheme.

The non-continuous rotation scheme samples specify the gyro outputs at appointed positions. As shown in the first graph in Figure 2, the vector OA points towards the observer i.e., position 1; the vector OG denotes the gyro sensitive axis; the vector ON point to north direction and *ψ* indicates the azimuth of the observer against the north direction. The angle between the observer and the current gyro axis is denoted as $\gamma_{1}$. As the gyro rotates non-continuously around the shaft by $360^{\circ }$ in *n* equal angular increments, we obtain the angle $\gamma_{i}$ (1 ≤ *i* ≤ *n*) and the gyro outputs at each position from 1 to *n*. The azimuth is achieved by fitting the gyro outputs at these positions using the azimuth measurement model. The following section describes our model in detail.

## 3. Modeling of the Gyro Outputs

The gyro detects the projection of the earth rotation rate on its sensitive axis and outputs its value. In this section, our mathematical model describing the relationship between the gyro outputs and the azimuth at each appointed position, i.e., position 1 to *n*, is proposed. Using this model, the azimuth is easily obtained from the gyro outputs. SubSection 3.1 proposes an azimuth measuring model in ideal condition. Since the azimuth measurement result is strongly affected by the gyro misalignment and the shaft misalignment, Subsection 3.2 presents a new measuring model with the former element taken into account, and Subsection 3.3 further improves this model by taking both the affecting elements into consideration.

### 3.1. Gyro Outputs Modeling Under Ideal Conditions

**Figure 3:**
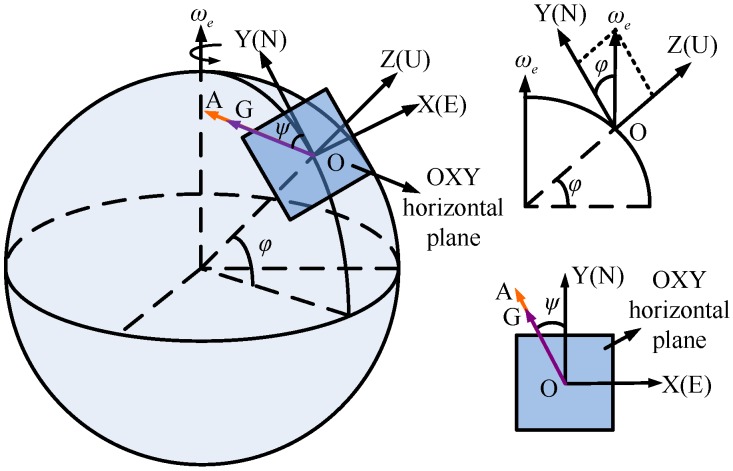
The east, north, up geographical coordinate system.

The aim of the north finder is to detect the azimuth *ψ* between the observer (first position of the gyro) and the north direction. Under ideal conditions (without the gyro and shaft misalignments), the azimuth measurement is performed in a local horizontal plane with the gyro axis kept parallel to the horizontal plane. In Figure 3, the sphere is the earth which rotates around itself with an angular rate $\omega_{e}$. The north finder is placed at point O with latitude *φ*. OXYZ is the geographical coordinate system, where the axes OX, OY, and OZ coincide with east, north, and up direction, respectively. Under ideal conditions, the gyro axis OG is parallel to the OXY horizontal plane at each rotation position *i*. At position 1, the gyro axis OG coincides with the direction OA.

It is clear from Figure 3 that the earth rotation angular velocity *ω* can be expressed in the geographical coordinate system as a column vector:

(1) $$\omega =\left[\begin{array}{c} 0\\ \omega_{e}\cos\varphi \\ \omega_{e}\sin\varphi \end{array}\right]$$

where $\omega_{e}$ is the magnitude of the earth angular velocity, with $\omega_{e}=15.041$${}^{\circ }/h$.

In order to detect the azimuth *ψ*, the earth angular velocity (i.e., the column vector *ω*) is rotated counterclockwise by *ψ* around the OZ axis. Using the right hand rule (all the following rotation matrices follow these rule), the rotation matrix is given by

(2) $$R_{Z}\left(\psi \right)=\left[\begin{array}{ccc} \cos\psi & -\sin\psi & 0\\ \sin\psi & \cos\psi & 0\\ 0 & 0 & 1 \end{array}\right]$$

It should be noted that in our system, the OXYZ coordinate system is fixed, while the vector *ω* is rotated. Then the rotated vector $\omega_{o}$ is obtained by using the matrix multiplication $R_{Z}\left(\psi \right)$*ω*:

(3) $$\omega_{o}=R_{Z}\left(\psi \right)\omega =\left[\begin{array}{c} -\omega_{e}\cos\varphi \sin\psi \\ \omega_{e}\cos\varphi \cos\psi \\ \omega_{e}\sin\varphi \end{array}\right]$$

Here, the projection of the vector $\omega_{o}$ on the OXY horizontal plane coincides with OA. When the gyro axis coincides with OA, the gyro output at this position is given by

(4) $$\omega_{og}=\omega_{o}\left(2\right)=\omega_{e}\cos\varphi \cos\psi +e_{b}+e_{r},$$

where $e_{b}$ and $e_{r}$ denote the gyro bias error and the gyro random error, respectively.

At a specific latitude, the azimuth *ψ* is achieved from Equation (4) if the gyro output $\omega_{og}$ is known. In practice, however, since only one gyro output is used, it requires the high bias stability of the gyro. Otherwise the small angular rate $\omega_{e}\cos\varphi \cos\psi$ will be too small compared to the gyro bias error $e_{b}$. Therefore, it is necessary to sample the gyro outputs at several positions. Hence, a multi-position non-continuous rotation north finding scheme is used in our system. As described in Figure 2, the gyro is rotated counterclockwise by $\gamma_{i}$ around axis OZ from position 1 to position *n*. We assume that the vector $\omega_{o}$ is rotated along side with the gyro. The corresponding rotation matrix is

(5) $$R_{Z}\left(\gamma_{i}\right)=\left[\begin{array}{ccc} \cos\gamma_{i} & -\sin\gamma_{i} & 0\\ \sin\gamma_{i} & \cos\gamma_{i} & 0\\ 0 & 0 & 1 \end{array}\right].$$

At position *i*, the vector $\omega_{r}$ is obtained by multiplying $R_{Z}\left(\gamma_{i}\right)$ by $\omega_{o}$:

(6) $$\omega_{r}=R_{Z}\left(\gamma_{i}\right)\omega_{o}=\left[\begin{array}{c} -\omega_{e}\cos\varphi \sin\left(\gamma_{i}+\psi \right)\\ \omega_{e}\cos\varphi \cos\left(\gamma_{i}+\psi \right)\\ \omega_{e}\sin\varphi \end{array}\right].$$

The projection of $\omega_{r}$ on the gyro axis at position *i* hence becomes

(7) $$\omega_{rgi}=\omega_{r}\left(2\right)=\omega_{e}\cos\varphi \cos\left(\gamma_{i}+\psi \right)+e_{b}+e_{r}.$$

Equation (7) is the mathematical model of the gyro outputs against azimuth at position *i* *without* taking the gyro and shaft misalignments into consideration.

### 3.2. Gyro Outputs Modeling with Gyro Misalignment

**Figure 4:**
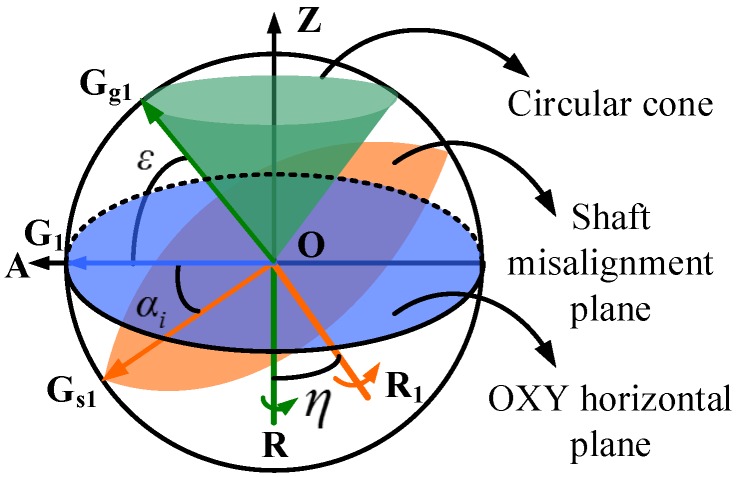
The gyro axis’s trajectory with the gyro misalignment or the shaft misalignment when the shaft is rotated counterclockwise around itself for 360${}^{\circ }$ (the sphere is not the earth, but is used to facilitate understanding of the misalignments).

Conversely, in the north finding system, the gyro misalignment [4] and the shaft misalignment [33] usually do exist and corrupt the azimuth measurement result. As shown in Figure 4 (the sphere is in this case not the earth, but used to facilitate the understanding of the misalignments), under ideal conditions, the shaft is aligned vertically to the horizontal plane and is represented here as the vector OR. The gyro sensitive axis OG lies in the horizontal plane and points in the observer direction. When the shaft rotates, the gyro axis always remains in the horizontal plane, i.e., the blue part. If the gyro is misaligned, there exist an angle *ε* between the gyro sensitive axis OG_g_ and the horizontal plane. It is defined as the gyro misalignment angle. As the shaft OR rotates, the trajectory of OG_g_ forms a circular cone (the green part) and *ε* remains the same at each rotation position. If the shaft is misaligned, the condition becomes complex. Assuming OR_1_ is the misaligned shaft, the orange plane will be the moving trajectory of the gyro sensitive axis OG_s_ as it rotates. Here, *η* is the angle between OR and OR_1_ and is defined as the shaft misalignment angle, and $\alpha_{i}$ is used to denote the angle of the gyro axis to the OXY horizontal plane. Unlike the case when the gyro is misaligned, the angle between the gyro axis OG_s_ and the OXY horizontal plane is different at different rotation positions. Since the characteristics of the gyro misalignment and the shaft misalignment are different, the two misalignments will be discussed separately, and eventually a generalized gyro outputs model will be presented. The gyro axis outputs model with only the gyro misalignment (*ε* ≠ 0, *η* = 0) is firstly presented. Then the model with also the shaft misalignment (*ε* = 0 or *ε* ≠ 0, *η* ≠ 0) is proposed.

In this part, *η* = 0 is assumed, i.e., only the gyro misalignment angle *ε* is considered. The gyro misalignment angle is obtained by rotating the gyro axis counterclockwise by -ε around the X axis. Likewise, we rotate the vector $\omega_{r}$ counterclockwise by -ε around the X axis, and then a new vector $\omega_{g}$ is obtained. Now the gyro outputs are equal to the projection of the vector $\omega_{g}$ on the gyro axis. The rotation matrix is

(8) $$R_{X}\left(-\varepsilon \right)=\left[\begin{array}{ccc} 1 & 0 & 0\\ 0 & \cos\varepsilon & \sin\varepsilon \\ 0 & -\sin\varepsilon & \cos\varepsilon \end{array}\right]$$

The vector $\omega_{g}$ is obtained by multiplying $R_{X}\left(-\varepsilon \right)$ by $\omega_{r}$:

(9) $$\omega_{g}=R_{X}\left(-\varepsilon \right)\omega_{r}=\left[\begin{array}{c} -\omega_{e}\cos\varphi \sin\left(\gamma_{i}+\psi \right)\\ \omega_{e}\cos\varphi \cos\left(\gamma_{i}+\psi \right)\cos\varepsilon +\omega_{e}\sin\varphi \sin\varepsilon \\ -\omega_{e}\cos\varphi \cos\left(\gamma_{i}+\psi \right)\sin\varepsilon +\omega_{e}\sin\varphi \cos\varepsilon \end{array}\right]$$

Thus the earth rotation rate detected by the gyro axis is equal to the projection of $\omega_{g}$ on the gyro axis:

(10) $$\omega_{ggi}=\omega_{g}\left(2\right)=\omega_{e}\cos\varphi \cos\left(\gamma_{i}+\psi \right)\cos\varepsilon +\omega_{e}\sin\varphi \sin\varepsilon +e_{b}+e_{r}$$

### 3.3. Gyro Outputs Modeling with Shaft Misalignment

In this part, the shaft misalignment condition (*η* ≠ 0) will be explained. The gyro axis is obtained by rotating the gyro axis counterclockwise by $-\alpha_{i}$ around the X axis. Similarly, we rotate the vector $\omega_{r}$ counterclockwise by $-\alpha_{i}$ around the X axis, and then a new vector $\omega_{s}$ is obtained. Now the gyro outputs are equal to the projection of the vector $\omega_{s}$ on the gyro axis. The rotation matrix is

(11) $$R_{X}\left(-\alpha_{i}\right)=\left[\begin{array}{ccc} 1 & 0 & 0\\ 0 & \cos\alpha_{i} & \sin\alpha_{i}\\ 0 & -\sin\alpha_{i} & \cos\alpha_{i} \end{array}\right]$$

The vector $\omega_{s}$ is obtained by multiplying $R_{X}\left(-\alpha \right)$ by $\omega_{r}$:

(12) $$\omega_{s}=R_{X}\left(-\alpha_{i}\right)\omega_{r}=\left[\begin{array}{c} -\omega_{e}\cos\varphi \sin\left(\gamma_{i}+\psi \right)\\ \omega_{e}\cos\varphi \cos\left(\gamma_{i}+\psi \right)\cos\alpha_{i}+\omega_{e}\sin\varphi \sin\alpha_{i}\\ -\omega_{e}\cos\varphi \cos\left(\gamma_{i}+\psi \right)\sin\alpha_{i}+\omega_{e}\sin\varphi \cos\alpha_{i} \end{array}\right]$$

Thus, the earth rotation rate detected by the gyro axis is equal to the projection of $\omega_{s}$ on the gyro axis:

(13) $$\omega_{sgi}=\omega_{s}\left(2\right)=\omega_{e}\cos\varphi \cos\left(\gamma_{i}+\psi \right)\cos\left(\alpha_{i}\right)+\omega_{e}\sin\varphi \sin\left(\alpha_{i}\right)+e_{b}+e_{r}$$

**Figure 5:**
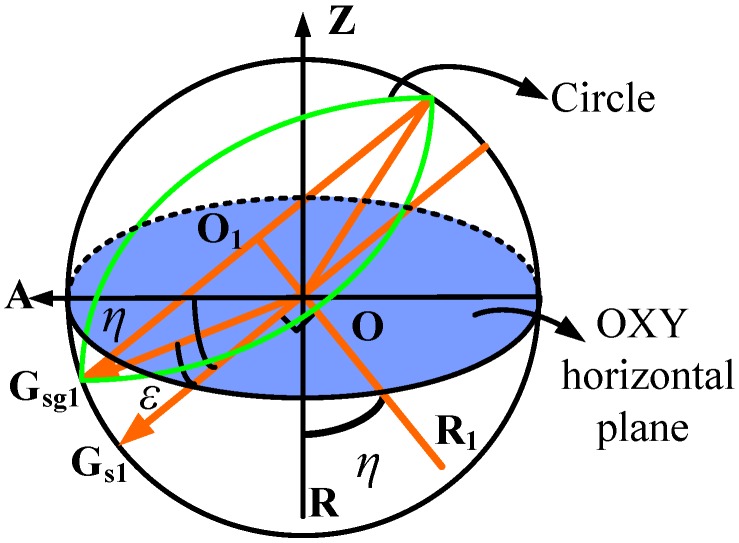
The gyro axis’s trajectory with the shaft misalignment and the gyro misalignment when the shaft is rotated counterclockwise around the shaft for 360${}^{\circ }$ (the sphere is not the earth, but used to facilitate understanding of the misalignments).

As shown in Figure 4, the value of $\alpha_{i}$ varies at different rotation positions *i* when *η* ≠ 0. Hence, the calculation of $\alpha_{i}$ is discussed. Based on the value of *ε* (*ε* = 0, *ε* ≠ 0), $\alpha_{i}$ is divided into two cases. As shown in Figure 5, the first case is *ε* = 0: OG_*s*1_ denotes the corresponding gyro axis at position 1, and $\alpha_{1}$ = *η*. The trajectory of gyro axis under this condition (*η* ≠ 0, *ε* = 0) is a plane. The second case is *ε* ≠ 0: the corresponding gyro axis is OG_*sg*1_, the angle G_*sg*1_OG_*s*1_ is *ε*, and $\alpha_{1}$ = *η* -ε. The trajectory of the gyro axis under this condition (*η* ≠ 0, *ε* ≠ 0) is a circular cone.

Here, the second case is discussed. The first case is achieved by defining *ε* = 0. The gyro axis at position *i* is OG_*sgi*_, and OG_*sgi*_ is defined as a unit vector, and then point G_*sg*1_ is located at (0, cos(*η*-ε), −sin(*η*-ε)). Point G_*sgi*_’s trajectory is a circle with its center O_1_ at (0, sin*ε* sin*η*, sin*ε*cos*η*), and point G_*sgi*_’s trajectory forms the shaft misalignment plane. The normal vector of the shaft misalignment plane is given by

(14) $$\overset{⇀}{\;n}=(0,\sin\eta ,\cos\eta )$$

Based on Equation (14) and point G_*sg*1_, the shaft misalignment plane equation equals

(15) (sinη)y+(cosη)z=sinε

In the rotating process, if the shaft O_1_R_1_ is rotated $\gamma_{i}$ counterclockwise around itself, so does O_1_G_*sg*1_. Assuming point G_*sgi*_ = ($x_{i}$, $y_{i}$, $z_{i}$), then OG_*sgi*_ is a unit vector, point G_*sgi*_ is on the shaft misalignment plane, and the angle between vector O_1_G_*sg*1_ and vector O_1_G_*sgi*_ is $\gamma_{i}$. Based on these conditions, the following set of equations about point G_*sgi*_ is thus obtained:

(16) xi2+yi2+zi2=1(sinη)yi+(cosη)zi=sinεcosγi=(cosηcosε(yi-sinηsinε)-sinηcosε(zi-cosηsinε))cosηcosε(yi-sinηsinε)-sinηcosε(zi-cosηsinε))cosε2cosε2

The coordinate of G${}_{sgi}$ is found by solving Equation (16):

(17) $$\left\{\begin{array}{c} x_{i}=-\cos\varepsilon \sin\gamma_{i}\\ y_{i}=\sin\eta \sin\varepsilon +\cos\eta \cos\varepsilon \cos\gamma_{i}\\ z_{i}=\cos\eta \sin\varepsilon -\sin\eta \cos\varepsilon \cos\gamma_{i} \end{array}\right.$$

Therefore, cos $\alpha_{i}$ is equal to the cosine of the vectors OG${}_{sgi}$ and OA${}_{i}$ (A${}_{i}$ is in OXY plane, and A${}_{i}$ = (−sin$\gamma_{i}$, cos$\gamma_{i}$, 0)):

(18) $$\cos\alpha_{i}=\sin{\gamma_{i}}^{2}\cos\varepsilon +\cos\gamma_{i}\left(\sin\eta \sin\varepsilon +\cos\eta \cos\varepsilon \cos\gamma_{i}\right)$$

Substituting Equation (18) into Equation (13), the gyro axis outputs model with the shaft and gyro misalignments is achieved.

(19) $$\left\{\begin{array}{c} \omega_{sgi}=\omega_{e}\cos\varphi \cos\left(\gamma_{i}+\psi \right)\cos\left(\alpha_{i}\right)+\omega_{e}\sin\varphi \sin\left(\alpha_{i}\right)+e_{b}+e_{r}\\ \cos\alpha_{i}=\sin{\gamma_{i}}^{2}\cos\varepsilon +\cos\gamma_{i}\left(\sin\eta \sin\varepsilon +\cos\eta \cos\varepsilon \cos\gamma_{i}\right)\\ \sin\alpha_{i}=\sqrt{1-\cos{\alpha_{i}}^{2}} \end{array}\right.$$

Equation (19) is simplified to Equation (10) when *η* = 0, and Equation (19) is reduced to Equation (7) when *ε* and *η* are both 0.

## 4. Estimate Azimuth Uncertainty

In order to estimate the measurement uncertainty of the north finding system, each critical measurement component should be identified and its uncertainty must be quantified. The parameters in the north finder system include the gyro bias error, the gyro random error, the number of measurement points *n*, the encoder uncertainty $\delta_{\gamma }$, the gyro misalignment angle *ε*, and the shaft misalignment angle *η*. Combining the gyro outputs model and the theory of propagation of uncertainty, these parameters are analyzed separately, and finally the overall azimuth uncertainty is presented.

Initially, the gyro measurement uncertainty is analyzed. According to Equation (19), the derivative of $\omega_{sgi}$ to *ψ* with *η* = *ε* = 0 is

(20) $$\frac{\partial \omega_{sgi}}{\partial \psi }=-\omega_{e}\cos\varphi \sin\left(\gamma_{i}+\psi \right)$$

The gyro bias is a nonzero output when the input is zero. Usually, the bias error is very small in a short period time when the temperature is stable. The gyro random error is a fluctuating output when the input is the same. Here we take the gyro bias error $e_{b}$ and the gyro random error $e_{r}$ as gyro measurement uncertainty [5]. At position *i*, the gyro measurement uncertainty’s effect on *ψ*’s uncertainty is described as

(21) $$\sigma_{\psi \omega_{sgi}}=\frac{\partial \psi }{\partial \omega_{sgi}}\sigma_{\omega }$$

where $\sigma_{\omega }$ is the gyro measurement uncertainty. In one circle measurement from position 1 to position *n*, the total azimuth uncertainty $\sigma_{\psi \omega sg}$ caused by the gyro measurement uncertainty is

(22) $$\sigma_{\psi \omega_{sg}}=\sqrt{\sum_{i=1}^{n}{\left(\frac{\partial \psi }{\partial \omega_{sg}}\right)}^{2}{\sigma_{\omega }}^{2}}$$

In Figure 2, the rotation angle is equal at every time in one circle, and the number of samples acquired *n* covers exactly a complete period [34], and then we have

(23) $$\left\{\begin{array}{c} \sum_{i=1}^{n}\cos\gamma_{i}=\sum_{i=1}^{n}\sin\gamma_{i}=\sum_{i=1}^{n}\sin\gamma_{i}\cos\gamma_{i}=0\\ \sum_{i=1}^{n}{\left(\cos\gamma_{i}\right)}^{2}=\sum_{i=1}^{n}{\left(\sin\gamma_{i}\right)}^{2}=\frac{n}{2} \end{array}\right.$$

Substituting Equations (20) and (23) into Equation (22), the azimuth uncertainty $\sigma_{\psi \omega_{sg}}$ is

(24) $$\sigma_{\psi \omega_{sg}}=\sqrt{\frac{2}{n}}\frac{\sigma_{\omega }}{\omega_{e}\cos\varphi }$$

**Figure 6:**
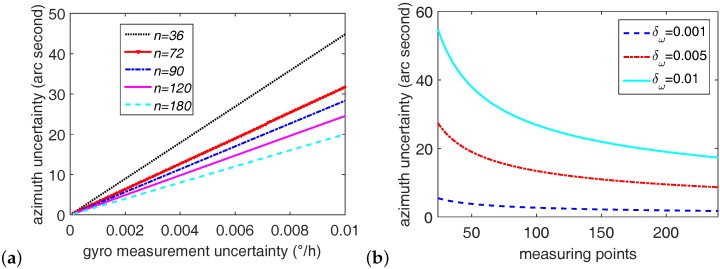
The relationship of the azimuth uncertaintyagainst the gyro random drift and the measuring points. (**a**) The azimuth uncertainty against random drift at various points *n*; (**b**) The azimuth uncertainty against random drift at various values of $\sigma_{\omega }$.

In Equation (24), $\omega_{e}$ = $15.041{\;}^{\circ }$/h and *φ* is a constant in specified latitude (for instance, *φ* = $43.8{\;}^{\circ }$ in Changchun, China). As shown in Figure 6a (arc second is used to denote the unit of azimuth uncertainty, and 1 arc second = 1” = $1/3600{\;}^{\circ }$), increasing the number of measurement points *n* and reducing the measurement uncertainty $\sigma_{\omega }$ will lower the azimuth uncertainty. However, it is noted that azimuth uncertainty decreases slowly when the $\sigma_{\omega }$ is quite small (see Figure 6b). In particular, when $\sigma_{\omega }$ = $0.005{\;}^{\circ }$/h, the azimuth uncertainty decreases from 15 arc second (*n* = 70) to 10 arc second (*n* = 240); when $\sigma_{\omega }$ = $0.001{\;}^{\circ }$/h, the azimuth uncertainty decreases from 3 arc second (*n* = 70) to 2 arc second (*n* = 240). Therefore, a proper number of measurement points should be chosen according to the required azimuth precision and the measurement time. In addition, the latitude affects the azimuth uncertainty too. Based on Equation (24), the azimuth uncertainty gradually decreases as the latitude decreases from high to low.

Subsequently, the encoder measurement uncertainty is analyzed. According to Equation (19), the azimuth uncertainty caused by the encoder measurement uncertainty $\sigma_{\gamma }$ at position *i* is

(25) $$\sigma_{\psi \gamma_{i}}=\sqrt{{\left(\frac{\partial \psi }{\partial \gamma_{i}}\right)}^{2}{\sigma_{\gamma }}^{2}}=\sqrt{{\left(\frac{\partial \psi }{\partial \omega_{sgi}}\frac{\partial \omega_{sgi}}{\partial \gamma_{i}}\right)}^{2}{\sigma_{\gamma }}^{2}}$$

Substituting Equation (23) into Equation (25), in one circle measurement the overall azimuth uncertainty caused by the encoder measurement uncertainty with *η* = *ε* = 0 is

(26) $$\sigma_{\psi \gamma }=\sigma_{\gamma }\sqrt{\sum_{i=1}^{n}{\left(\frac{\partial \psi }{\partial \omega_{sgi}}\frac{\partial \omega_{sgi}}{\partial \gamma_{i}}\right)}^{2}}=\sigma_{\gamma }$$

According to Equation (26), the encoder uncertainty $\sigma_{\gamma }$ has a significant effect on the azimuth results, and the encoder uncertainty will propagate to the azimuth uncertainty directly. Therefore, a high precision encoder is required for a high precision north finder.

Next, the gyro misalignment’s effect is analyzed. According to Equation (19), gyro outputs with *η* = 0 equal

(27) $$\omega_{\mathrm{sg}i}=\omega_{e}\cos\varphi \cos\left(\gamma_{i}+\psi \right)\cos\varepsilon +\omega_{e}\sin\varphi \sin\varepsilon$$

Here *ε* is a constant for the assembled gyro, $\omega_{e}$ = $15.041{\;}^{\circ }$/h, and *φ* is constant in a specified latitude. Thus Equation (27) can be rewritten as

(28) $$\left\{\begin{array}{c} \omega_{\mathrm{sg}i}=C_{1}\cos\left(\gamma_{i}+\psi \right)+C_{2}\\ C_{1}=\omega_{e}\cos\varphi \cos\varepsilon ,C_{2}=\omega_{e}\sin\varphi \sin\varepsilon \end{array}\right.$$

Here C${}_{1}$ and C${}_{2}$ are constants, hence *ε* has no effect on the azimuth uncertainty when *η* = 0.

Finally, the shaft misalignment’s effect on the azimuth results is analyzed. The azimuth uncertainty caused by the shaft misalignment is

(29) $$\sigma_{\psi \eta }=\sigma_{\eta }\sqrt{\sum_{i=1}^{n}{\left(\frac{\partial \psi }{\partial \omega_{sgi}}\frac{\partial \omega_{sgi}}{\partial \eta }\right)}^{2}}$$

Substituting Equation (19) into Equation (29), $\sigma_{\psi \eta }$ is obtained as follows, where the symbols $a_{1}$, $a_{2}$, $a_{3}$, $a_{4}$, $a_{5}$, $a_{6}$, $b_{1}$, $b_{2}$ are introduced to simplify the equation:

(30) σψη=σηncosφ2(a1+a2)+sinφ2((a3+a4+a5)(a3+a4+a5)a6a6)ncosφ2(b1+b2)a1=cosη2sinε2(0.125+0.25cosψ2)a2=sinη2cosε2(0.0625+0.25cosψ2)a3=sinη2cosη2(0.375sinε4+0.2734cosε4)a4=sinη2cosη(0.0782cosε4-0.125sinε2cosε2)a5=0.0625cosε2cosη2sinε2+0.0234cosε4sinη2+0.3125sinε2cosε2a6=1-(0.375cosε2+0.25cosε2cosη+0.5sinη2sinε2+0.375cosη2cosε2)b1=sinη2sinε2(0.125+0.25sinψ2)+cosη2cosε2(0.0625+0.25sinψ2)b2=0.25cosψ2cosε2+0.0625cosε2+0.125cosε2cosη

**Figure 7:**
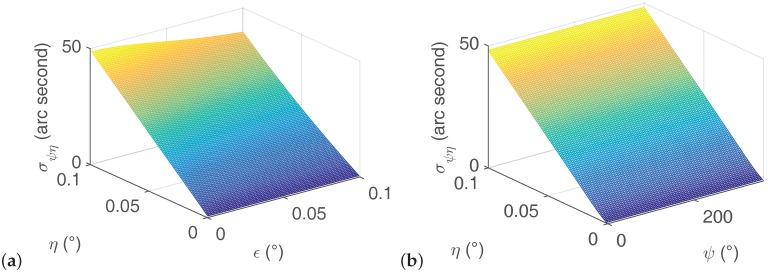
Azimuth uncertainty against shaft misalignment. (**a**) Azimuth uncertainty against shaft misalignment when *ε*∈ [$0.001^{\circ }$, $0.1^{\circ }$] and *ψ* = $45^{\circ }$; (**b**) Azimuth uncertainty against shaft misalignment when $\psi \in [0^{\circ },360^{\circ }]$ and *ε* = $0.01^{\circ }$.

According to Equation (30), the relationship of the azimuth uncertainty against the shaft misalignment is shown in Figure 7. It is clear from Figure 7a that the azimuth uncertainty $\sigma_{\psi \eta }$ increases as the shaft misalignment angle *η* grows; however, as the gyro misalignment angle *ε* increases, the azimuth uncertainty slightly falls. When the shaft misalignment angle *η* equals zero, the azimuth uncertainty remains at zero regardless of the value of *ε*. Differently, the azimuth value *ψ* shows no effect on azimuth uncertainty (see Figure 7b). Usually, the gyro misalignment angle *ε* is very small and measuring it is troublesome. Therefore, a much better choice for achieving a lower azimuth uncertainty is to reduce the shaft misalignment angle.

Based on the theory of propagation of uncertainty, the total azimuth uncertainty $\sigma_{\psi }$ is expressed as:

(31) $$\sigma_{\psi }=\sqrt{{\sigma_{\psi \omega_{sg}}}^{2}+{\sigma_{\psi \gamma }}^{2}+{\sigma_{\psi \eta }}^{2}}$$

According to Equation (31), in order to improve the azimuth measurement precision, the following procedures should be conducted:

1. Constrain the gyro bias error and the gyro random error. Regarding to the former, on one hand, selecting a high precision gyro usually attains a low bias error; on the other hand, using filtering approaches [20,24,32] is an alternative method to reduce the gyro bias error. For the gyro random error, a simple and effective method is to average a large number of gyro outputs at each rotation position. Other filtering approaches can be found in [21,22,24,25] .
2. Increase the number of measurement points (ensure that the north finding time is less than the time scale of the bias instability [4]).
3. Execute the azimuth measurement at a low latitude area. When the north finding experiment is executed at the equator ($\varphi =0^{\circ }$), all the earth rotation rate is detected by the gyro sensitive axis. In contrast, the north finding task is impossible to achieve when *φ* = 90${}^{\circ }$. Generally, the north finding experiment should be conducted away from the Antarctic Circle and the Arctic Circle.
4. Improve the encoder measurement precision. In the multi-position north finding scheme, a high precise rotary platform and a corresponding high precision encoder are prerequisites to achieve high precision azimuth results.
5. Align the shaft to make the shaft misalignment angle as small as possible. As described in Equation (28), the gyro misalignment angle has no effect on the azimuth results when shaft misalignment angle is 0. However, when a shaft misalignment exists, both the shaft misalignment angle and the gyro misalignment angle corrupt the gyro outputs, as shown in Figure 7a. Therefore, the best choice is to align the shaft.

The criteriafor the selection of the system components, including the rate gyro, the inclinometer and the encoder, are listed as follows:

1. Building a north finding system with azimuth uncertainty of $\sigma_{\psi }$ (Equation (31)) is the goal. Through rough calculation (distribute azimuth uncertainty), the value of $\sigma_{\psi \omega_{sg}}$, $\sigma_{\psi \gamma }$ and $\sigma_{\psi \eta }$ (Equation (31)) are determined.
2. Based on the value of $\sigma_{\psi \omega_{sg}}$ and Equation (24), the gyro bias error and the gyro random error are decided, and then the specified rate gyro can be selected.
3. Combining the value of $\sigma_{\psi \gamma }$ and Equation (26), the encoder uncertainty is calculated, thus the encoder is selected.
4. According the value of $\sigma_{\psi \eta }$ and Equation (29), the shaft misalignment angle is calculated, thus the shaft misalignment angle measurement instrument can be chosen. In practice, we usually align this angle to a very small value. The details of shaft misalignment angle calculation and alignment process refer to [35].

## 5. Implementation of a High Precision Robust Gyro North Finder

**Figure 8:**
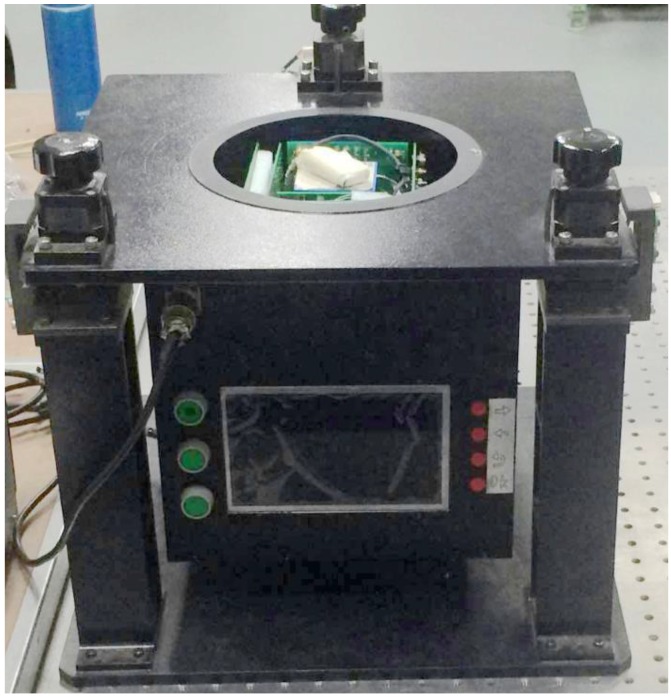
Gyro north finder setup.

To evaluate the proposed north finding method, a north finder setup is built as shown in Figure 8. Currently, depending on the performance specifications of the gyros, MEMS gyros are used in consumer area, ring laser gyros are used in tactical area, resonant optical gyros, fiber optic gyros and dynamically tuned gyros are used in navigational area [5]. In order to achieve high precision azimuth measurement, a dynamically tuned gyro in navigational grade is used in our setup. The dynamically tuned gyro in this setup has a bias error of $0.003{\;}^{\circ }$/h. Through filtering, the gyro random error is found to be $0.002{\;}^{\circ }$/h. Hence, the gyro measurement uncertainty is $0.005{\;}^{\circ }$/h. At the same latitude (*φ* = $43.8{\;}^{\circ }$), all experiments are carried out using the scheme in Figure 2. At each position *i*, the gyro stays for 2 s and is then automatically rotated counterclockwise by 360/*n*${}^{\circ }$ to position *i* + 1 by the motor.

### 5.1. Gyro Outputs Modeling with Shaft Misalignment

As described in Figure 7, the shaft misalignment will corrupt the gyro outputs. In order to achieve high precision azimuth result, it is essential to measure the shaft misalignment angle *η* between the practical shaft OR_1_ and the vertical shaft OR, and to adjust the practical shaft to a vertical state. The shaft misalignment angle measurement procedure includes two steps using the inclinometer in our system [35]. Firstly, the inclinometer outputs are obtained at position 1, and $\theta_{X1}$, $\theta_{Y1}$ indicate the inclinometer X+ axis and the Y+ axis outputs respectively. Then the inclinometer is rotated counterclockwise by $180^{\circ }$ around the shaft to position *i*, and $\theta_{X2}$, $\theta_{Y2}$ indicate the inclinometer X+ axis and the Y+ axis outputs respectively. Generally, $\left(\theta_{X2}-\theta_{X1}\right)/2$ and $\left(\theta_{Y2}-\theta_{Y1}\right)/2$ are small angles (less than $1^{\circ }$), and then the relationship of *η* against the inclinometer outputs is

(32) $$\eta \approx \arctan\sqrt{{\left(\tan\frac{\theta_{X2}-\theta_{X1}}{2}\right)}^{2}+{\left(\tan\frac{\theta_{Y2}-\theta_{Y1}}{2}\right)}^{2}}.$$

The shaft misalignment angle is adjusted by regulating the three knobs. *η* = 0 is acquired when $\theta_{X1}$ = $\theta_{X2}$, $\theta_{Y1}$ = $\theta_{Y2}$. The resolution of the inclinometer in our experiment is 0.0005${}^{\circ }$ (NS-5/P2), and the standard deviation of the shaft alignment angle reaches at 0.001${}^{\circ }$. Using the approaches in [35], a shaft alignment precision of 0.003${}^{\circ }$ is achieved.

**Figure 9:**
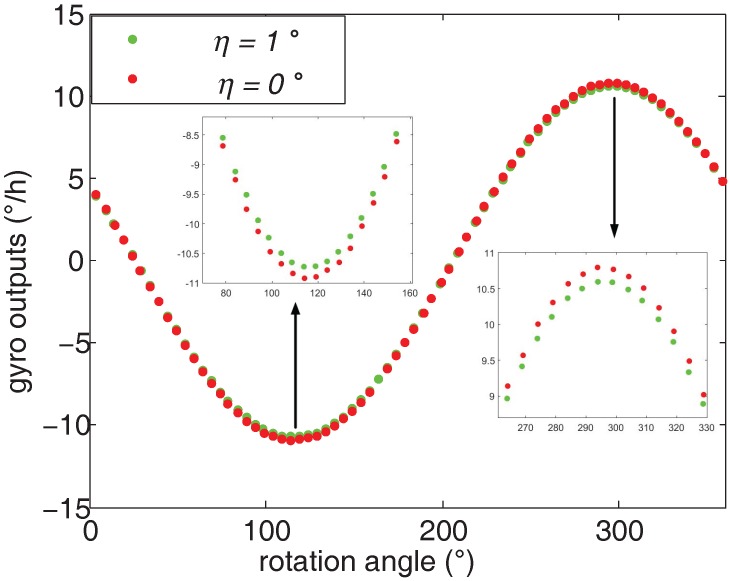
Comparison of the gyro outputs when the shaft is misaligned and aligned.

Two comparison experiments are conducted to evaluate the shaft misalignment’s effect on azimuth measurement results. The first experiment is conducted at *η* = $1^{\circ }$, and the second one is operated at *η* = $0^{\circ }$. The rotation points *n* are 72 in both experiments and their gyro outputs are shown in Figure 9. Ideally, the curve of the gyro outputs from position 1 to position *n* is a standard sinusoidal. However, when the shaft is misaligned, the practical gyro outputs deviates from the ideal gyro outputs. As shown in Figure 9, the gyro outputs of *η* = $1^{\circ }$) is bigger than those of *η* = $0^{\circ }$ in the troughs; in contrast, the gyro outputs of *η* = $1^{\circ }$) is smaller than those of *η* = $0^{\circ }$ in the peaks.

**Figure 10:**
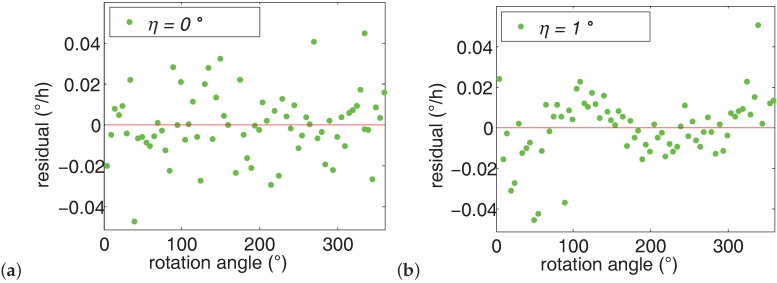
Gyro outputs residual. (**a**) aligned state. (**b**) misaligned state.

The function of $\omega_{sgi}=A\cos\gamma_{i}+B\sin\gamma_{i}+C$ is used to fit the gyro outputs. By subtracting the fitting results and the gyro outputs, the gyro outputs residual of *η* = $0^{\circ }$ and *η* = $1^{\circ }$ are obtained as shown in Figure 10. Naturally, the residual is randomly located around the line of *x* = 0. However, it is clear from Figure 10 that the locations of the residual of *η* = $1^{\circ }$ does not fit this pattern. This is due to the fact that the gyro outputs of *η* = $1^{\circ }$ deviate from the sinusoidal gyro outputs using sine wave fitting. Therefore, it is strongly recommended to align the shaft to vertical state before north finding. The following experiments are all conducted in shaft aligned state.

### 5.2. Azimuth Measurement Results

Increasing the number of measurement points in one circle measurement lowers the azimuth uncertainty but takes more time. This is a trade-off of azimuth uncertainty and measurement time. Therefore, a proper number of measurement points should be chosen according to Equation (31) to attain a required azimuth precision. In our north finder system, $\sigma_{\gamma }$ = $0.001^{\circ }$ and *η* = $0.003^{\circ }$. Based on Equations (26) and (29), $\sigma_{\psi \gamma }$ = 3.6” and $\sigma_{\psi \eta }$ = 0.7”. Substituting $\omega_{e}$, $\sigma_{\omega }$ and *φ* by $15.041^{\circ }$, $0.005^{\circ }$ and $43.8^{\circ }$ respectively, and specifying *n* into Equation (24), $\delta_{\psi \omega e4s}$ is achieved. Finally, the overall azimuth uncertainty $\delta_{\psi }$ is calculated using Equation (31). Here, two criteria assist us to select the number of measurement points. The first one is to calculate the specified *n* from the given required azimuth uncertainty according to Equation (31). The second one is to ensure that the number of measurement points *n* large enough to attain desirable fitting results. Based on these two criteria, the minimum value of *n* is set at 72 in our system, and the north finding results of *n* = 72, *n* = 90, *n* = 120 and *n* = 180 are given.

When *n* is set at 72, 10 repeated azimuth measurement experiments are successively conducted based on the working process in Figure 2. For each run, the gyro outputs and the corresponding encoder outputs at the 72 positions are recorded. $\omega_{sgi}=A\cos\gamma_{i}+B\sin\gamma_{i}+C$ is then used to fit the gyro outputs. The ten fitting results are given in Table 1.

**Figure 11:**
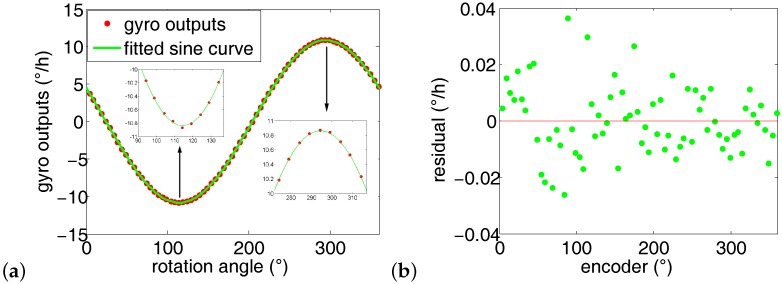
Gyro outputs fitting results when the number of measurement points *n* = 72. (**a**) Fitting curve when *n* = 72; (**b**) Residual of the corresponding fitting curve.

Averaging the 10 runs’ azimuth results in Table 1, the average azimuth is 65.5378${}^{\circ }$. The standard deviation is 33”. In each run, the maximum of the sum of residual square is less than 0.15 × 0.001 (${}^{\circ }$/h)${}^{2}$. The first run’s gyro outputs is plotted in Figure 11. As expected, the gyro maximum output is 10.83${}^{\circ }$/h, pointing north; the minimum output is −10.83${}^{\circ }$/h, pointing south. The gyro outputs fit the sine wave quite well (see Figure 11a), and the maximum residual is less than 0.04${}^{\circ }$/h.

Table 2 gives the 10 groups of azimuth measurement results when *n* is set as 90.

**Figure 12:**
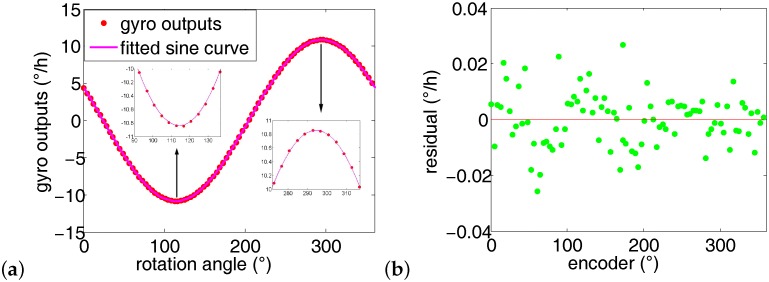
Gyro outputs fitting results when the number of measurement points *n* = 90. (**a**) Fitting curve when *n* = 90; (**b**) Residual of the corresponding fitting curve.

According to Table 2, the average azimuth is 65.5467${}^{\circ }$, and the standard deviation is 25 arc second. The maximum of the sum of residual square in each run is less than 0.9 ×$0.0001({\;}^{\circ }$/h)${}^{2}$, which is 0.6 × 0.0001 (${}^{\circ }$/h)${}^{2}$ smaller than the former 10 runs when *n* is set at 72. Similarly, the first run’s experiment data in Table 2 are chosen to illustrate the azimuth fitting results, as shown in Figure 12. The maximum residual is less than 0.03${}^{\circ }$/h, about 25% lower than that of the first 10 runs.

Thirdly, setting *n* = 120, Table 3 gives the 10 groups of azimuth measurement results.

**Figure 13:**
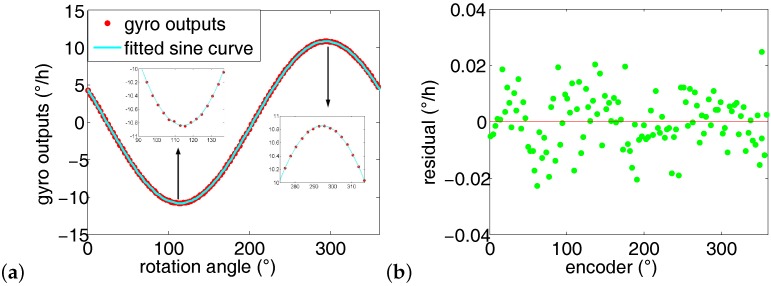
Gyro outputs fitting results when the number of measurement points *n* = 120. (**a**) Fitting curve when *n* = 120; (**b**) Residual of the corresponding fitting curve.

As listed in Table 3, the average azimuth is 65.5504${}^{\circ }$, and the standard deviation is 22”. The maximum of the sum of residual square in each run is less than 0.9 × 0.0001 (${}^{\circ }$/h)${}^{2}$. Figure 13 shows the fitting results of the 1-th group experiment data in Table 3. The maximum residual is less than 0.025${}^{\circ }$/h, which is 37.5% lower than the first ten 10 runs.

Finally, Table 4 gives the 10 groups of azimuth measurement results when *n* = 180.

**Figure 14:**
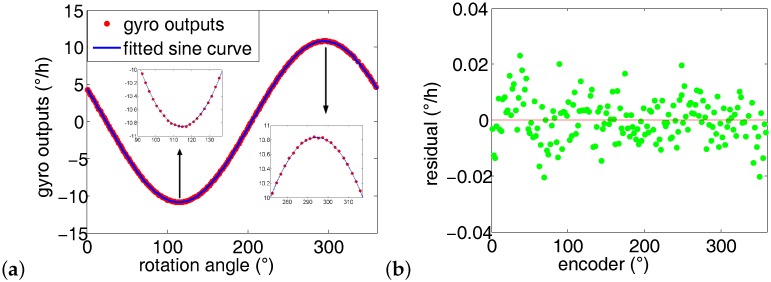
Gyro outputs fitting results when the number of measurement points *n* = 180. (**a**) Fitting curve when *n* = 180; (**b**) Residual of the corresponding fitting curve.

According to Table 4, the average azimuth is 65.5445${}^{\circ }$, and the standard deviation is 18 arc second. The maximum of the sum of residual square in each run is less than 0.82 ×$0.0001({\;}^{\circ }$/h)${}^{2}$. The first group experiment data in Table 4 are chosen to illustrate the azimuth fitting process, as shown in Figure 14. The maximum residual is less than 0.023${}^{\circ }$/h, about 40% smaller than that of the first 10 runs.

Based on Equation (31), the simulated azimuth uncertainties are 19”, 17”, 15” and 13” when *n* are 72, 90, 120 and 180 respectively. As shown in Table 1, Table 2, Table 3 and Table 4, the experimental azimuth uncertainties are 33”, 25”, 22” and 18” arc second accordingly. The differences between the azimuth uncertainties and the simulated azimuth uncertainties are 14” (*n* = 72), 8”(*n* = 90), 7”(*n* = 120) and 5”(*n* = 180). Clearly, the experimental azimuth uncertainties are closer and closer to the simulated azimuth uncertainties as *n* goes up. Equation (31) is obtained according to Equation (19), and Equation (19) is a continuous function. However, in our experiment, the number of measurement points *n* is a limited number, i.e., the gyro outputs are digitized. Hence, the larger the value of *n* is, the better the fitting results are. In our experiment, the rotation time from position *i* to position *i* + 1 is about 0.2 s, and the sampling time at each measurement position is 2 s, so 2.2 s are taken at each measurement. Therefore, the measuring time are about 158 s, 198 s, 264 s, 396 s when *n* are 72, 90, 120 and 180 respectively. Increasing the number of measurement points leads to higher precision but longer measuring time.

## 6. Conclusions

In this paper we propose a multi-position non-continuous rotation gyro north finding method to measure the azimuth between the observer and north direction. We proposed a mathematical model for gyro outputs at each rotation position. Our model concerns the key factors related to the azimuth results, including the gyro bias error, the gyro random error, scale-factor error, encoder precision, the number of measurement points, the gyro misalignment angle, and the shaft misalignment angle. Combining the gyro outputs model and the theory of propagation of uncertainty, the optimized azimuth measurement scheme is achieved. According to this, the corresponding approaches to attain high precision azimuth results are provided. Finally, the azimuth uncertainty of 18” (equals to 0.087 mrad) is obtained in our system. Currently, the azimuth uncertainty of the reported north finding system [4] and the product on the market are single digit mrad. It is noted that the specifications of the gyros has a significant effect on the azimuth uncertainty according to Equation (31), therefore, high performance gyros, e.g., navigational grade gyros, achieve better azimuth measurement results than consumer grade gyros. This paper provides systematic theory for analyzing the details of the gyro north finder scheme from simulation to implementation. It is useful and valuable to both applied researchers in academia and advanced practitioners in industry.

## Figures and Tables

**Table 1:** Azimuth measurement results with *n* = 72.

Run	Azimuth (${}^{\circ }$)	Sum of Residual Square (×0.001${}^{\circ }$)
1	65.5378	0.1484
2	65.5484	0.1047
3	65.5324	0.1249
4	65.5523	0.1021
5	65.5542	0.1045
6	65.5472	0.0911
7	65.5452	0.0668
8	65.5588	0.1059
9	65.5554	0.074
10	65.5341	0.1337

**Table 2:** Azimuth measurement results with *n* = 90.

Run	Azimuth (${}^{\circ }$)	Sum of Residual Square (×0.0001${}^{\circ }$)
1	65.5411	0.6271
2	65.5225	0.894
3	65.5497	0.606
4	65.5558	0.8603
5	65.5524	0.5651
6	65.5571	0.6942
7	65.5538	0.6859
8	65.5568	0.5812
9	65.5427	0.5754
10	65.5523	0.603

**Table 3:** Azimuth measurement results with *n* = 120.

Run	Azimuth (${}^{\circ }$)	Sum of Residual Square (×0.0001${}^{\circ }$)
1	65.5411	0.8966
2	65.5225	0.7253
3	65.5497	0.6616
4	65.5558	0.6652
5	65.5524	0.4259
6	65.5571	0.7311
7	65.5538	0.6436
8	65.5568	0.6466
9	65.5427	0.6887
10	65.5523	0.4927

**Table 4:** Azimuth measurement results with *n* = 180.

Run	Azimuth (${}^{\circ }$)	Sum of Residual Square (×0.0001${}^{\circ }$)
1	65.5457	0.5889
2	65.5277	0.8186
3	65.5376	0.6666
4	65.5488	0.5508
5	65.5513	0.723
6	65.5401	0.5321
7	65.5392	0.5979
8	65.5493	0.5302
9	65.5402	0.8117
10	65.5451	0.5569
